# Identification and characterization of dermatophyte species and strains with PCR amplification

**DOI:** 10.3892/etm.2014.1785

**Published:** 2014-06-13

**Authors:** GUOFANG LIU, CHENGHUA HE, HAIBIN ZHANG

**Affiliations:** 1 College of Veterinary Medicine, Nanjing Agricultural University, Nanjing, Jiangsu 210095, P.R. China; 2 Department of Animal Husbandry and Veterinary Medicine, Jiangsu Polytechnic College of Agriculture and Forestry, Zhenjiang, Jiangsu 212400, P.R. China

**Keywords:** dermatophyte, polymerase chain reaction, microsatellite primer, non-transcribed spacer

## Abstract

The aim of the present study was to use two polymerase chain reaction (PCR) methods, with (GACA)_4_ and non-transcribed spacer (NTS) as primers, to identify and characterize dermatophyte isolates from dogs and cats to a species and strain level. A total of 45 isolates from nine dermatophyte species were collected from pet dogs and cats and subjected to PCR amplification with the microsatellite primer (GACA)_4_. Dermatophyte strains of three of the same species collected from four cities were subjected to PCR amplification with the NTS primer set. These two PCR methods were applied to identify and characterize the dermatophyte isolates to a species and strain level. Regional differences among the strain specificities were also examined. The results from PCR with (GACA)_4_ demonstrated that strains from the same species produced similar PCR product band patterns. In addition, these patterns differed among species, indicating that (GACA)_4_ primer-based PCR was able to distinguish between the various dermatophyte species. By contrast, dermatophyte isolates and/or strains within the same species revealed various band patterns with NTS-based PCR. In addition, the results indicated that regional differences contributed to the variations in PCR product band patterns. Therefore, the results of the present study indicate that the NTS-based PCR method is efficient in distinguishing dermatophytes to the strain level, while a combination of (GACA)_4_ and NTS primer-based PCR methods is able to clarify dermatophyte isolates to a species and strain level. The present study provides information concerning the identification of pathogenic fungi and the epidemiological characteristics of fungal skin diseases.

## Introduction

Fungal infections affect superficial keratinized tissues, including the skin, hair and nails, in humans and animals resulting in difficult-to-treat dermatosis. Fungi derived from pet dogs and cats, including dermatophytes (Deuteromycotina, Hyphomycetes, Hyphomycetales, Moniliaceae, *Trichophyton*, *Microsporum* and *Epidermophyton*), *Malassezia*, Saccharomycetes (mainly *Candida*) and non-dermatophyte molds (*Scopulariopsis*, *Aspergillus* and *Fusarium*), are also able to infect human skin (1). The routine identification and classification of these skin-infecting fungi is mainly based on clinical symptoms and the morphological and/or biochemical characteristics of the fungi. In recent years, molecular approaches, including pulsed-field gel electrophoresis, random amplified polymorphic DNA analysis, polymerase chain reaction (PCR) amplification using NTS and internal transcribed spacer (ITS) primers (2), nested-PCR, PCR-restriction fragment length polymorphism (RFLP) analysis, arbitrary primer PCR and ITS region sequencing (3), have been used for the identification of dermatophyte species and strains.

The simple repetitive oligonucleotide, (GACA)_4_, is a highly variable microsatellite that has been used as a PCR primer for the efficient identification of skin tinea infections and pathogenic *Candida* species (4). PCR using (GACA)_4_ has also been used for the classification and identification of human pathogenic fungi (5). In the present study, microsatellite (GACA)_4_ and non-transcribed spacer (NTS) primers were used to perform PCR amplification with the aim of identifying and characterizing dermatophyte isolates from dogs and cats to a species and strain level.

## Materials and methods

### Fungal strains

Pathogenic fungal strains were isolated from pet dogs and cats. In total, 45 strains were analyzed for species identification and characterization, including five strains from each of the following species: *Trichophyton rubrum* (*T. rubrum*), *Trichophyton mentagrophytes* (*T. mentagrophytes*), *Epidermophyton floccosum* (*E. floccosum*), *Microsporum canis* (*M. canis*), *Microsporum gypseum* (*M. gypseum*), *Candida albicans* (*C. albicans*), *Candida tropicalis* (*C. tropicalis*), *Candida glabrata* (*C. glabrata*) and *Candida parapsilosis* (*C. parapsilosis*).

For strain comparison, 54 strains of *T. rubrum*, 26 strains of *T. mentagrophytes* and 32 strains of *M. canis* were collected from four cities, namely Nanjing, Wuxi, Shanghai and Hangzhou in China.

The strains were cultured in Sabouraud dextrose agar medium at 27°C with a humidity of 95% in a mold incubator. Filamentous fungi were usually cultured for 2 weeks, whereas yeasts were cultured for 2–3 days.

### DNA extraction and purification

Fungal genomic DNA was extracted using the benzyl chloride extraction method and purified with phenol-chloroform as previously described (6,7). Growing fungi were harvested through filtration and washed three times with sterile saline. The samples were transferred to 1.5-ml microcentrifuge tubes and subjected to centrifugation at 5,700 × g at room temperature for 1 min. Next, 500 μl extraction buffer [100 mM Tris-HCl (pH 9.0) and 40 mM EDTA], 100 μl sodium dodecyl sulfate (10%) and 300 μl benzyl chloride (Sinochem Ningbo Chemicals Co., Ltd., Ningbo, China) were added. The mixture was vortexed and incubated at 50°C for 3 min with mild shaking. Following centrifugation at 6,000 × g at a temperature of 4°C for 10 min, the supernatant was collected in a new tube. Next, 300 μl sodium acetate (3 M) was added and the samples were mixed and centrifuged again at 6,000 × g at 4°C for 10 min. Following aspiration, the supernatant was transferred into a tube containing 500 μl isopropanol. The sample was stored at −70°C for 1 h and DNA was precipitated following centrifugation at 6,000 × g at 4°C for 10 min. Extracted DNA was treated with RNase A and then with phenol/chloroform/isoamyl alcohol (v:v:v, 25:24:1; all purchased from Sinochem Ningbo Chemicals Co., Ltd.). Following 2 or 3 centrifugations, DNA was precipitated with ice-cold pure ethanol, washed with 70% ethanol, air-dried and then resuspended in Tris-EDTA buffer for additional study. Yeast genomic DNA was prepared using a quick DNA extraction kit (Shanghai Huashun Bioengineering Co., Ltd., Shanghai, China), according to the manufacturer’s instructions.

### PCR amplification

PCR amplification was conducted in volumes of 100 μl containing 10 mM Tris-HCl (pH 9.0), 0.1% Triton X-100, 50 mM KCl, 1.5 mM MgCl_2_, 200 μM dATP, dCTP, dGTP and dTTP, 5 units *Taq* DNA polymerase (Takara Bio, Inc., Dalian, China), 2.5 μM primer and 20 ng template DNA. Primer sequences are shown in Table I (synthesized by Shanghai Yingweijie Co., Shanghai, China). PCR amplification was performed in a GeneAmp PCR System 9600 (Perkin-Elmer, Norwalk, CT, USA). Samples were first heated at 94°C for 4 min, followed by 35 cycles of 92°C for 1 min, 55°C for 1 min (NTS-1 primers) or 58°C for 1 min (NTS-2 primers) and 72°C for 2 min, prior to an extension step at 72°C for 10 min. For amplification using (GACA)_4_, PCR was carried out for 39 cycles of denaturation at 93°C for 1 min, annealing at 50°C for 1 min, and extension at 72°C for 1 min, followed by a final extension step at 72°C for 7 min. The products were electrophoresed in 2% agarose gels and detected using a gel imaging analysis system (Bio-Rad, Hercules, CA, USA).

### Statistical analysis

Data were analyzed using SPSS software, version 10.0 (SPSS, Inc., Chicago, IL, USA). Differences were compared with the χ^2^ test and P<0.05 was considered to indicate a statistically significant difference.

## Results

### Identification of dermatophyte species using (GACA)_4_ primer-based PCR

**Figure 1:**
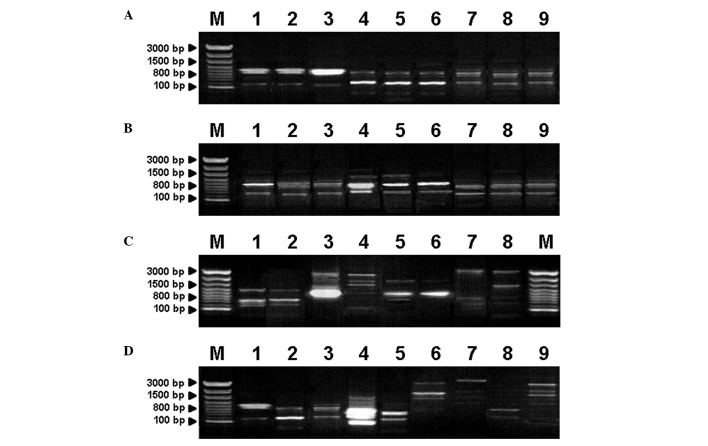
Identification of dermatophyte species using (GACA)_4_ primer-based PCR. PCR products of various dermatophyte isolates are shown. Lanes: M, molecular weight marker; (A) 1–3, *T. rubrum*; 4–6, *T. mentagrophytes*; 7–9, *M. canis*; (B) 1–3, *M. canis*; 4–6, *M. gypseum*; 7–9, *E. floccosum*; (C) 1 and 2, *C. albicans*; 3 and 4, *C. glabrata*; 5 and 6, *C. tropicalis*; 7 and 8, *C. parapsilosis*; (D) 1, *T. rubrum*; 2, *T. mentagrophytes*; 3, *M. canis*; 4, *M. gypseum*; 5, *E. floccosum*; 6, *C. albicans*; 7, *C. parapsilosis*; 8, *C. tropicalis*; 9, *C. glabrata*. PCR, polymerase chain reaction; *T. rubrum*, *Trichophyton rubrum; T. mentagrophytes, Trichophyton mentagrophytes; M. canis, Microsporum canis; M. gypseum, Microsporum gypseum; E. floccosum, Epidermophyton floccosum; C. albicans, Candida albicans; C. glabrata, Candida glabrata; C. tropicalis; Candida tropicalis; C. parapsilosis; Candida parapsilosis.*

PCR, with the short oligonucleotide (GACA)_4_ as a primer, was performed to identify and characterize dermatophytes. DNA products were determined by gel electrophoresis and image analysis. The results showed that the PCR product bands ranged between 300 and 3,000 bp (Fig. 1). Based on clinical phenotypic analysis, strains from the same species produced similar patterns, but these patterns changed from species to species (Fig. 1). These results indicate that (GACA)_4_ primer-based PCR is able to distinguish between various dermatophyte species, which may be useful for species identification.

### Characterization of dermatophyte strains using NTS-based PCR

**Figure 2:**
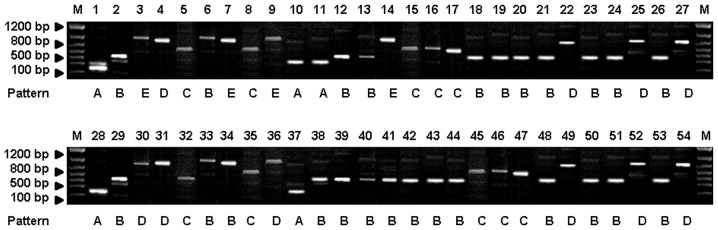
Characterization of 54 *T. rubrum* strains using PCR with the NTS-1 primer sets. Patterns were designated arbitrarily as A, B, C, D and E. Lanes: M, molecular weight marker; 1–54, 54 *T. rubrum* strains collected from four cities. PCR, polymerase chain reaction; NTS, non-transcribed spacer; *T. rubrum*, *Trichophyton rubrum.*

**Figure 3:**
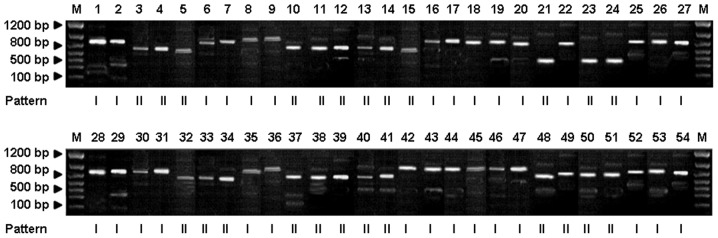
Characterization of 54 *T. rubrum* strains using PCR with NTS-2 primer sets. Patterns were designated arbitrarily as I and II. Lanes: M, molecular weight marker; 1–54, 54 *T. rubrum* strains collected from four cities. PCR, polymerase chain reaction; NTS, non-transcribed spacer; *T. rubrum*, *Trichophyton rubrum.*

To determine intraspecies variation and identify dermatophyte isolates to the strain level, PCR amplification was performed with NTS-1 and NTS-2 primer sets. The dermatophyte isolates were collected from four cities in China. As shown in Fig. 2, the NTS-1 amplification products from 54 *T. rubrum* strains were divided into five patterns (A, B, C, D and E) with typical fragment sizes of 250, 550, 750, 900 and 1,000 bp, respectively (Fig. 2). By contrast, the NTS-2 amplification products exhibited two band patterns (I and II) with typical fragment sizes of 500 and 450 bp, respectively (Fig. 3).

**Figure 4:**
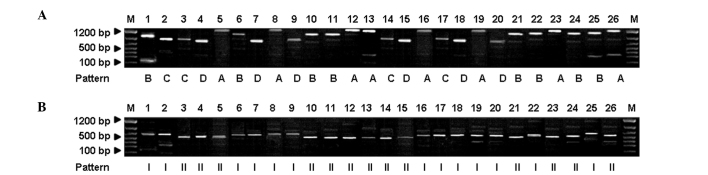
Characterization of 26 *T. mentagrophytes* strains using NTS-based PCR. PCR products with (A) NTS-1 primer sets (patterns were designated arbitrarily as A, B, C and D) and (B) NTS-2 primer sets (patterns were designated arbitrarily as I and II). Lanes: M, molecular weight marker; 1–26, 26 *T. mentagrophytes* strains collected from four cities. PCR, polymerase chain reaction; NTS, non-transcribed spacer; *T. mentagrophytes*, *Trichophyton mentagrophytes.*

**Figure 5:**
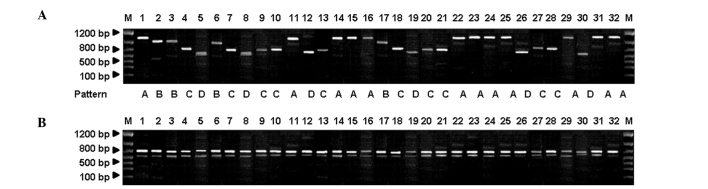
Characterization of 32 *M. canis* strains using NTS-based PCR. PCR products with (A) NTS-1 primer sets (patterns were designated arbitrarily as A, B, C and D) and (B) NTS-2 primer sets. Lanes: M, molecular weight marker; 1–32, 32 *M. canis* strains collected from four cities. PCR, polymerase chain reaction; NTS, non-transcribed spacer; *M. canis, Microsporum canis.*

The NTS-1-based PCR amplification products from the strains of *T. mentagrophytes* were divided into four patterns (A, B, C and D) with typical fragment sizes of 850, 900, 1,100 and 1,200 bp, respectively (Fig. 4A). In addition, the NTS-2-based PCR amplification products were divided into two patterns (I and II) with typical fragment sizes of 800 and 650 bp, respectively (Fig. 4B). For *M. canis*, the NTS-1 amplification products were divided into four patterns (A, B, C and D) with typical fragment sizes of 1,100, 1,000, 800 and 750 bp, respectively (Fig. 5A). However, the NTS-2 amplification products for the various strains exhibited the same profile, consisting of two clearly distinguishable bands of 800 and 650 bp (Fig. 5B). These results indicate that dermatophyte isolates and/or strains within the same species exhibit various band patterns with NTS-based PCR, indicating that this method may be a useful tool to identify dermatophytes to the strain level.

### Regional differences in the NTS-based PCR product band patterns from the same dermatophyte species

To investigate the regional differences among dermatophyte strains, NTS-based PCR band pattern percentages were analyzed. Intraspecies classification of the NTS-1-based PCR amplification band patterns of 54 *T. rubrum* strains is shown in Table II. In Nanjing and Wuxi, pattern B accounted for a relatively large proportion when compared with the other four band patterns. By contrast, patterns D and C were predominant in Hangzhou and Shanghai, respectively. For NTS-2-based PCR amplification, 30 of the 54 *T. rubrum* strains were classified as pattern I, accounting for 55.56%. The remaining 24 strains were classified as pattern II, accounting for 44.44% (Fig. 3). The majority of strains from Nanjing and Wuxi were classified as pattern I, while strains from Hangzhou and Shanghai were primarily classified as pattern II.

For *M. canis*, patterns A and C of the NTS-1-based PCR amplification product bands accounted for 37.50 and 31.25% of strains, respectively. There were no regional differences for *M. canis* in patterns A and C of the NTS-1-based PCR amplification product bands. The NTS-1 and NTS-2-based PCR amplification band patterns of *T. mentagrophytes* exhibited no statistically significant differences among the various regions, although the incidence of pattern I strains in Wuxi and Nanjing was slightly higher than that in the other locations. Therefore, these results indicate that regional differences contribute to variations in PCR product band patterns, indicating that NTS-based PCR may be efficient in distinguishing dermatophytes to the strain level.

## Discussion

At present, skin fungal classification and identification is primarily based on the clinical symptoms and characteristics of *in vitro* culture. However, this is a time-consuming process that is not able to identify dermatophyte strains. In addition, accuracy and precision is easily affected by culture conditions and environmental factors. In the present study, (GACA)_4_ and NTS primer-based PCR methods were applied to identify dermatophyte isolates to a species and strain level. The results revealed significant differences in the (GACA)_4_ primer-based PCR amplification band patterns among the tested 45 clinical dermatophyte isolates. Band patterns were clear with specific distributions, rendering them distinguishable. The PCR product band patterns of the nine species were similar to those described by Zhu *et al* (5), with an extra 1,500 bp fragment produced in amplification. However, further experiments are required to confirm whether this difference is attributed to the various origins of these dermatophyte strains.

The characteristics of ITS- and NTS-based PCR amplification patterns have been applied to study fungal species specificities (4,8). An ITS is a relatively conserved gene sequence involved in species specificity of dermatophytes (9). The 18, 5.8 and 25 S gene fragments of ITS1 and ITS2 from dermatophytes can be PCR-amplified using NTS 9 and ITS 6 as primers (10). The ITS regions of 37 *T. mentagrophytes* strains have been sequenced and divided into three homology groups and intraspecies specificity has also been analyzed. Jackson *et al* classified *T. rubrum* into 14 types using probes designed by the sequences of 18 S rDNA and ITS regions (11). The majority of strains fell into four types with evident polymorphisms. However, the methods used by Jackson *et al* were rather complex.

An NTS is a highly variable region; thus, its sequence is ideal for distinguishing between species and/or strains. Mochizuki *et al* divided *T. rubrum* into five types, according to the RFLP analysis of NTS (12). Jackson *et al* also confirmed the specificity, reproducibility and stability of NTS (11). A common method to distinguish between the species of *T. mentagrophytes* and *M. canis* uses ITS regions and random primers (13–16). Jackson *et al* amplified the ITS regions of 17 dermatophyte strains and digested the samples with *Mva*I. A total of 13 fragments were produced. One band was specifically produced in each of the nine fungi, including *M. canis*, *M. gypseum* and *T. mentagrophytes*, indicating that the *Mva*I digestion reaction only partly reflects the inter- and intraspecific specificities. Although NTS-2 has no specificity within the *M. canis* species, the specificities of ITS and NTS may be used to classify *T. mentagrophytes*, *M. canis* and *T. rubrum* derived from dogs and cats. ITS and NTS have been further demonstrated to be specific in the classification of pathogenic fungi (17). Despite the efficiency of DNA sequencing in fungal species classification, the complexity and high cost limits its application. RFLP analysis of ITS and NTS in rDNA is simple and the results are stable (18). This procedure is easily and widely applicable to the identification of fungal species and is important for the study of fungal epidemiology.

The results of the present study indicate that regional differences contribute to variations in PCR product bands of dermatophyte strains, and possess the potential to distinguish dermatophytes to the strain level. However, for *T. rubrum*, the pattern distribution in the current study was not consistent with the six-type classification by Fan *et al* (19) and the 14-type classification by Jackson *et al* (21). This discrepancy may be associated with sample insufficiency and the limited sampling regions. Furthermore, the NTS-based PCR amplification product band patterns of *T. mentagrophytes* and *M. canis* did not exhibit differences among regions as clearly as those in *T. rubrum*. Therefore, more samples are required to verify whether significant regional differences exist in the NTS-based PCR amplification product band patterns of these dermatophyte species.

In conclusion, using (GACA)_4_ and NTS as primers, PCR was accurately, conveniently and efficiently performed to clarify dermatophyte isolates to a species and strain level. The present study provides information concerning the identification of pathogenic fungi and the epidemiological characteristics of fungal skin diseases.

## Figures and Tables

**Table I:** PCR primer sets.

Regions	Primers	Sequences
NTS-1	TrNTSF-2	5′-ACC GTA TTA AGC TAG CGC TGC-3′
	TrNTSR-4	5′-TGC CAC TTC GAT TAG GAG GC-3′
NTS-2	TrNTSR-1	5′-CTC AGT CGA ACC GTG AGG C-3′
	TrNTSC-1	5′-CGA GAC CAC GTG ATA CAT GCG-3′

PCR, polymerase chain reaction; NTS, non-transcribed spacer.

**Table II:** Classification of NTS-1-based PCR amplification band patterns for *T. rubrum*.

Location	Pattern A, n (%)	Pattern B, n (%)	Pattern C, n (%)	Pattern D, n (%)	Pattern E, n (%)
Nanjing	1 (5.26)	12 (63.16)	2 (10.53)	2 (10.53)	2 (10.53)
Wuxi	3 (20.00)	8 (53.33)	1 (6.67)	1 (6.67)	2 (13.33)
Hangzhou	1 (8.33)	3 (25.00)	2 (16.67)	6 (50.00)	0 (0.00)
Shanghai	0 (0.00)	1 (12.5)	5 (62.50)	1 (12.50)	1 (12.5)
Total	5 (9.26)	24 (44.44)	10 (18.52)	10 (18.52)	5 (9.26)

NTS, non-transcribed spacer; PCR, polymerase chain reaction; *T. rubrum*, *Trichophyton rubrum*.
